# *N*-(2-Chloro­phen­yl)-4-hydr­oxy-2*H*-1,2-benzothia­zine-3-carboxamide 1,1-dioxide

**DOI:** 10.1107/S1600536809033972

**Published:** 2009-08-29

**Authors:** Waseeq Ahmad Siddiqui, Hamid Latif Siddiqui, Muhammad Azam, Masood Parvez, Umar Farooq Rizvi

**Affiliations:** a Department of Chemistry, University of Sargodha, Sargodha 40100, Pakistan; b Institute of Chemistry, University of the Punjab, Lahore 54590, Pakistan; c Institute of Biochemistry, University of Balochistan, Quetta, Pakistan; d Department of Chemistry, The University of Calgary, 2500 University Drive NW, Calgary, Alberta, Canada T2N 1N4

## Abstract

In the title compound, C_15_H_11_ClN_2_O_4_S, there are two independent mol­ecules in the asymmetric unit, in which the heterocyclic thia­zine rings in both mol­ecules adopt half-chair conformations. The conformations about the C—C and C—N bonds in the central C—C—N—C chain in both mol­ecules are all *EZ*. There are strong intra­molecular O—H⋯O and N—H⋯N hydrogen bonds resulting in graph-set patterns *S*(6) and *S*(5) for the oxo and amino rings, in addition to intra­molecular N—H⋯Cl inter­actions. In the crystal structure, mol­ecules are linked by inter­molecular O—H⋯O and N—H⋯O hydrogen bonds into chains along [100].

## Related literature

For details of the synthesis, see: Siddiqui *et al.* (2008 ▶). For background to benzothia­zine carboxamide derivatives as analgesic and anti-inflammatory agents, see: Myung *et al.* (2002 ▶); Shin *et al.* (2000 ▶); Banerjee & Sarkar (2002 ▶). For related structures, see: Siddiqui *et al.* (2006 ▶, 2007 ▶, 2008 ▶). Allen *et al.* (1987 ▶). For hydrogen-bond patterns and graph sets, see: Bernstein *et al.* (1994 ▶).
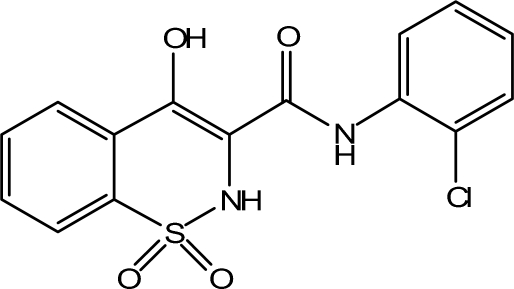


## Experimental

### 

#### Crystal data

C_15_H_11_ClN_2_O_4_S
*M* *_r_* = 350.77
Monoclinic, 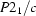

*a* = 10.077 (2) Å
*b* = 13.818 (3) Å
*c* = 21.426 (4) Å
β = 97.070 (13)°
*V* = 2960.8 (10) Å^3^
*Z* = 8
Mo *K*α radiation
μ = 0.42 mm^−1^
*T* = 200 K
0.16 × 0.14 × 0.12 mm

#### Data collection

Nonius KappaCCD diffractometer
Absorption correction: multi-scan (*SORTAV*; Blessing, 1997 ▶) *T*_min_ = 0.936, *T*_max_ = 0.951
12965 measured reflections
6732 independent reflections
5711 reflections with *I* > 2σ(*I*)
*R*_int_ = 0.031

#### Refinement

*R*[*F* ^2^ > 2σ(*F* ^2^)] = 0.041
*wR*(*F* ^2^) = 0.101
*S* = 1.05
6732 reflections
433 parameters
H atoms treated by a mixture of independent and constrained refinement
Δρ_max_ = 0.33 e Å^−3^
Δρ_min_ = −0.39 e Å^−3^

### 

Data collection: *COLLECT* (Hooft, 1998 ▶); cell refinement: *DENZO* (Otwinowski & Minor, 1997 ▶); data reduction: *SCALEPACK* (Otwinowski & Minor, 1997 ▶); program(s) used to solve structure: *SHELXS97* (Sheldrick, 2008 ▶); program(s) used to refine structure: *SHELXL97* (Sheldrick, 2008 ▶); molecular graphics: *ORTEP-3 for Windows* (Farrugia, 1997 ▶); software used to prepare material for publication: *SHELXL97*.

## Supplementary Material

Crystal structure: contains datablocks global, I. DOI: 10.1107/S1600536809033972/lh2886sup1.cif

Structure factors: contains datablocks I. DOI: 10.1107/S1600536809033972/lh2886Isup2.hkl

Additional supplementary materials:  crystallographic information; 3D view; checkCIF report

## Figures and Tables

**Table 1:** Hydrogen-bond geometry (Å, °)

*D*—H⋯*A*	*D*—H	H⋯*A*	*D*⋯*A*	*D*—H⋯*A*
O3—H3*O*⋯O4	0.83 (3)	1.89 (3)	2.612 (2)	144 (3)
O3—H3*O*⋯O4^i^	0.83 (3)	2.33 (3)	2.854 (2)	122 (2)
O7—H7*O*⋯O8	0.89 (3)	1.81 (3)	2.607 (2)	147 (2)
O7—H7*O*⋯O8^ii^	0.89 (3)	2.46 (3)	2.964 (2)	116 (2)
N1—H1*N*⋯O8^ii^	0.87 (2)	2.06 (2)	2.911 (2)	164 (2)
N2—H2*N*⋯N1	0.82 (2)	2.24 (2)	2.700 (2)	116 (2)
N2—H2*N*⋯Cl1	0.82 (2)	2.47 (2)	2.930 (2)	116 (2)
N3—H3*N*⋯O4^i^	0.89 (2)	2.07 (2)	2.912 (2)	157 (2)
N4—H4*N*⋯N3	0.88 (2)	2.23 (2)	2.692 (2)	113 (2)
N4—H4*N*⋯Cl2	0.88 (2)	2.41 (2)	2.934 (2)	119 (2)

Symmetry codes: (i) 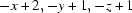; (ii) 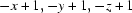.

**Fig. 1.**
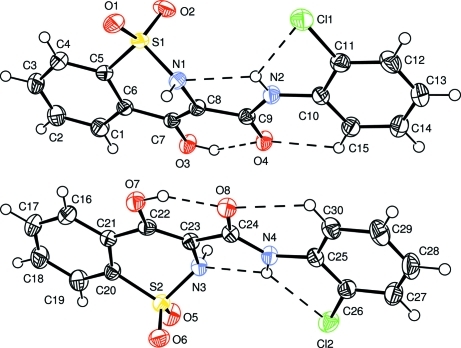
ORTEP-3 (Farrugia, 1997) drawing of molecules A and B in the asymmetric unit of (I) with displacement ellipsoids plotted at 50% probability level; intramolecular interactions have been drawn with dashed lines.

**Fig. 2.**
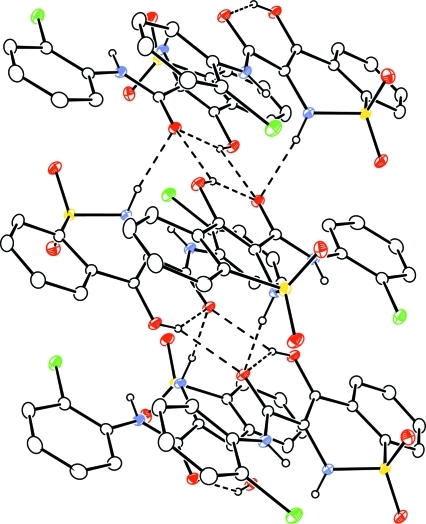
Part of the crystal structure of (I) with hydrogen bonds shown as dashed lines.

## supplementary crystallographic information

### Comment

Benzothiazine carboxamide derivatives are important due to their role as analgesic and anti-inflammatory agents (Myung *et al.*, 2002). These compounds belong to the oxicam class of non-steroidal anti-inflammatory drugs (NSAIDs) and are free from steroidal side-effects. However, these are ulcerogenic in behavior to varying degrees (Shin *et al.*, 2000). Besides great therapeutic potential, these compounds are very motivating polyfunctional heterocycles by virtue of their dynamic structural features (Banerjee & Sarkar, 2002). The search for more effective anti-inflammatory agents has led us to the synthesis of new agents using readily available starting material following facile routes to yield several products (Siddiqui *et al.*, 2006; Siddiqui *et al.*, 2007). In continuation of this program, we required the title compound, (I), to act as a nucleus for a variety of biologically active 1,2-benzothiazine-1,1-dioxide derivatives. Herein, we report the crystal structure of the title compound.

There are two molecules and in the asymmetric unit of the title compound (Fig. 1); the molecules containing S1 and S2 are referred to as molecules A and B, respectively. The bond lengths and bond angles in both molecules of (I) are within normal ranges (Allen *et al.*, 1987) and agree well with the corresponding bond lengths and bond angles of its *N*-methyl analogues (Siddiqui *et al.*, 2008).

The heterocyclic thiazine rings in both molecules of (I) adopt half-chair conformations wherein S1 and N1 are displaced by 0.439 (4) and -0.291 (3) Å, respectively, from the plane defined by C5/C6/C7/C8 atoms in molecule A and S2 and N3 displaced by -0.463 (4) and 0.284 (4) Å, respectively, from the plane defined by C20/C21/C22/C23 atoms in the molecule B. The puckering parameters (Cremer & Pople, 1975) in molecules A and B are: *Q* = 0.477 (2) and 0.489 (2) Å, θ = 118.2 (2) and 117.7 (2)° and φ = 203.8 (3) and 202.9 (3)°, respectively. Similar conformations of the thiazine ring have been reported in the structures related to (I) (Siddiqui *et al.*, 2008).

The conformations about the bonds C8–C9 and C9–N2 in molecule A and the bonds C23–C24 and C24–N4 in molecule B are all *EZ*, as determined by the strong intramolecular hydrogen bonds O3–H3O···O4 and N2–H2N···N1 in molecule A and O7–H7O···O8 and N4—H4N···N3 in molecule B resulting in graph set patterns S(6) and S(5) for the oxo and amino rings, respectively (Bernstein *et al.*, 1994). The intramolecular hydrogen bonds of the types N–H···Cl and C–H···O are also present in both molecules which represent S(5) and S(6) motifs, respectively. The structure is stabilized by intermolecular hydrogen bonds of the types O–H···O and N–H···O (details of H-bonding geometry have been provided in Table 1 and depicted in Fig. 2). The central atoms N2/O4/C8/C9/C10 in molecule A and N4/O8/C23/C24/C25 in molecule B are individually planar with maximum deviations of atoms from the planes being 0.0086 (16) and 0.0127 (14) Å for C9 and N4, respectively.

### Experimental

The method of preparation of the title compound has already been reported (Siddiqui *et al.*, 2006; Siddiqui *et al.*, 2007). Crystal of (I) suitable for X-ray crystallographic study were obtained by slow evaporation of its methanol solution at 313 K.

### Refinement

Though all the H atoms could be distinguished in the difference Fourier map the H-atoms were included at geometrically idealized positions and refined in riding-model approximation with the following constraints: C—H distances were set to 0.95 and 0.99 Å and N–H distance = 0.88 Å with *U*_iso_(H) = 1.2*U*_eq_(C/N). The final difference map was free of any chemically significant features.

### Crystal data

**Table:**

C_15_H_11_ClN_2_O_4_S	*F*(000) = 1440
*M**_r_* = 350.77	*D*_x_ = 1.574 Mg m^−^^3^
Monoclinic, *P*2_1_/*c*	Melting point = 491–492 K
Hall symbol: -P 2ybc	Mo *K*α radiation, λ = 0.71073 Å
*a* = 10.077 (2) Å	Cell parameters from 12965 reflections
*b* = 13.818 (3) Å	θ = 2.8–27.5°
*c* = 21.426 (4) Å	µ = 0.42 mm^−^^1^
β = 97.070 (13)°	*T* = 200 K
*V* = 2960.8 (10) Å^3^	Block, colorless
*Z* = 8	0.16 × 0.14 × 0.12 mm

### Data collection

**Table:**

Nonius KappaCCD diffractometer	6732 independent reflections
Radiation source: fine-focus sealed tube	5711 reflections with *I* > 2σ(*I*)
graphite	*R*_int_ = 0.031
ω and φ scans	θ_max_ = 27.5°, θ_min_ = 2.8°
Absorption correction: multi-scan (*SORTAV*; Blessing, 1997)	*h* = −13→13
*T*_min_ = 0.936, *T*_max_ = 0.951	*k* = −17→17
12965 measured reflections	*l* = −27→27

### Refinement

**Table:**

Refinement on *F*^2^	Primary atom site location: structure-invariant direct methods
Least-squares matrix: full	Secondary atom site location: difference Fourier map
*R*[*F*^2^ > 2σ(*F*^2^)] = 0.041	Hydrogen site location: inferred from neighbouring sites
*wR*(*F*^2^) = 0.101	H atoms treated by a mixture of independent and constrained refinement
*S* = 1.05	*w* = 1/[σ^2^(*F*_o_^2^) + (0.032*P*)^2^ + 2.78*P*] where *P* = (*F*_o_^2^ + 2*F*_c_^2^)/3
6732 reflections	(Δ/σ)_max_ = 0.001
433 parameters	Δρ_max_ = 0.33 e Å^−^^3^
0 restraints	Δρ_min_ = −0.39 e Å^−^^3^

### Special details

**Table:**

Geometry. All e.s.d.'s (except the e.s.d. in the dihedral angle between two l.s. planes) are estimated using the full covariance matrix. The cell e.s.d.'s are taken into account individually in the estimation of e.s.d.'s in distances, angles and torsion angles; correlations between e.s.d.'s in cell parameters are only used when they are defined by crystal symmetry. An approximate (isotropic) treatment of cell e.s.d.'s is used for estimating e.s.d.'s involving l.s. planes.
Refinement. Refinement of *F*^2^ against ALL reflections. The weighted *R*-factor *wR* and goodness of fit *S* are based on *F*^2^, conventional *R*-factors *R* are based on *F*, with *F* set to zero for negative *F*^2^. The threshold expression of *F*^2^ > σ(*F*^2^) is used only for calculating *R*-factors(gt) *etc*. and is not relevant to the choice of reflections for refinement. *R*-factors based on *F*^2^ are statistically about twice as large as those based on *F*, and *R*- factors based on ALL data will be even larger.

### Fractional atomic coordinates and isotropic or equivalent isotropic displacement parameters (Å^2^)

**Table:**

	*x*	*y*	*z*	*U*_iso_*/*U*_eq_	
Cl1	0.53894 (6)	0.85003 (4)	0.44704 (3)	0.03897 (14)	
Cl2	1.01898 (6)	0.42658 (4)	0.72988 (3)	0.03888 (14)	
S1	0.56632 (5)	0.56337 (4)	0.29991 (2)	0.02616 (11)	
S2	0.88023 (5)	0.18009 (4)	0.55861 (2)	0.02725 (12)	
O1	0.42471 (14)	0.56870 (11)	0.28299 (7)	0.0345 (3)	
O2	0.65098 (16)	0.63267 (11)	0.27504 (7)	0.0376 (4)	
O3	0.91579 (14)	0.44058 (11)	0.40450 (7)	0.0306 (3)	
H3O	0.942 (3)	0.475 (2)	0.4358 (13)	0.046*	
O4	0.90157 (13)	0.58281 (10)	0.48371 (7)	0.0299 (3)	
O5	1.01519 (15)	0.14585 (11)	0.56604 (8)	0.0394 (4)	
O6	0.79336 (16)	0.15325 (11)	0.60351 (7)	0.0361 (3)	
O7	0.55675 (14)	0.35003 (11)	0.46691 (7)	0.0316 (3)	
H7O	0.552 (3)	0.405 (2)	0.4885 (13)	0.047*	
O8	0.62498 (14)	0.48103 (10)	0.55245 (6)	0.0285 (3)	
N1	0.59401 (16)	0.56899 (12)	0.37671 (8)	0.0246 (3)	
H1N	0.527 (2)	0.5434 (17)	0.3934 (11)	0.030*	
N2	0.72054 (17)	0.68431 (12)	0.46688 (8)	0.0273 (4)	
H2N	0.653 (2)	0.6924 (17)	0.4419 (11)	0.033*	
N3	0.88570 (16)	0.29841 (12)	0.55636 (8)	0.0256 (3)	
H3N	0.956 (2)	0.3194 (17)	0.5386 (11)	0.031*	
N4	0.81507 (17)	0.45318 (12)	0.62112 (8)	0.0259 (3)	
H4N	0.881 (2)	0.4116 (17)	0.6302 (11)	0.031*	
C1	0.7962 (2)	0.32702 (15)	0.30815 (10)	0.0331 (5)	
H1	0.8737	0.3034	0.3333	0.040*	
C2	0.7414 (2)	0.27512 (17)	0.25567 (11)	0.0384 (5)	
H2	0.7817	0.2161	0.2452	0.046*	
C3	0.6289 (2)	0.30844 (17)	0.21865 (10)	0.0380 (5)	
H3	0.5912	0.2716	0.1834	0.046*	
C4	0.5707 (2)	0.39514 (16)	0.23257 (10)	0.0339 (5)	
H4	0.4937	0.4185	0.2069	0.041*	
C5	0.6264 (2)	0.44760 (14)	0.28448 (9)	0.0255 (4)	
C6	0.73784 (19)	0.41364 (14)	0.32404 (9)	0.0248 (4)	
C7	0.79336 (18)	0.46879 (14)	0.38005 (8)	0.0227 (4)	
C8	0.72638 (18)	0.54292 (14)	0.40405 (9)	0.0235 (4)	
C9	0.78939 (19)	0.60425 (14)	0.45506 (9)	0.0235 (4)	
C10	0.75144 (19)	0.75747 (14)	0.51204 (9)	0.0256 (4)	
C11	0.6697 (2)	0.83982 (15)	0.50801 (10)	0.0298 (4)	
C12	0.6902 (2)	0.91379 (16)	0.55174 (11)	0.0391 (5)	
H12	0.6331	0.9687	0.5486	0.047*	
C13	0.7943 (3)	0.90723 (17)	0.60010 (11)	0.0411 (5)	
H13	0.8091	0.9575	0.6304	0.049*	
C14	0.8764 (2)	0.82715 (17)	0.60399 (11)	0.0391 (5)	
H14	0.9487	0.8233	0.6369	0.047*	
C15	0.8559 (2)	0.75196 (16)	0.56085 (10)	0.0326 (5)	
H15	0.9129	0.6970	0.5647	0.039*	
C16	0.6273 (2)	0.19136 (17)	0.39968 (10)	0.0375 (5)	
H16	0.5581	0.2327	0.3811	0.045*	
C17	0.6553 (3)	0.10598 (19)	0.36988 (11)	0.0454 (6)	
H17	0.6051	0.0894	0.3309	0.054*	
C18	0.7550 (3)	0.04472 (18)	0.39617 (11)	0.0430 (6)	
H18	0.7738	−0.0131	0.3749	0.052*	
C19	0.8279 (2)	0.06714 (16)	0.45352 (11)	0.0361 (5)	
H19	0.8957	0.0247	0.4722	0.043*	
C20	0.7998 (2)	0.15269 (14)	0.48302 (9)	0.0266 (4)	
C21	0.7005 (2)	0.21657 (14)	0.45671 (9)	0.0259 (4)	
C22	0.67419 (19)	0.30822 (14)	0.48818 (9)	0.0244 (4)	
C23	0.76116 (18)	0.34608 (14)	0.53550 (9)	0.0233 (4)	
C24	0.72824 (18)	0.43260 (14)	0.57014 (9)	0.0239 (4)	
C25	0.81369 (19)	0.52796 (14)	0.66608 (9)	0.0260 (4)	
C26	0.9062 (2)	0.52284 (15)	0.72014 (9)	0.0300 (4)	
C27	0.9109 (2)	0.59287 (18)	0.76655 (10)	0.0398 (5)	
H27	0.9747	0.5883	0.8030	0.048*	
C28	0.8223 (3)	0.66939 (18)	0.75945 (11)	0.0430 (6)	
H28	0.8241	0.7174	0.7913	0.052*	
C29	0.7307 (2)	0.67612 (16)	0.70588 (12)	0.0403 (5)	
H29	0.6705	0.7293	0.7011	0.048*	
C30	0.7255 (2)	0.60632 (15)	0.65903 (10)	0.0331 (5)	
H30	0.6625	0.6118	0.6224	0.040*	

### Atomic displacement parameters (Å^2^)

**Table:**

	*U*^11^	*U*^22^	*U*^33^	*U*^12^	*U*^13^	*U*^23^
Cl1	0.0360 (3)	0.0319 (3)	0.0464 (3)	0.0097 (2)	−0.0055 (2)	0.0013 (2)
Cl2	0.0392 (3)	0.0409 (3)	0.0342 (3)	0.0065 (2)	−0.0048 (2)	0.0003 (2)
S1	0.0264 (2)	0.0246 (2)	0.0261 (2)	0.00015 (19)	−0.00253 (18)	0.00546 (19)
S2	0.0291 (3)	0.0255 (2)	0.0263 (2)	0.00761 (19)	−0.00002 (18)	−0.00181 (19)
O1	0.0272 (8)	0.0375 (8)	0.0357 (8)	0.0038 (6)	−0.0079 (6)	0.0052 (7)
O2	0.0430 (9)	0.0289 (8)	0.0412 (8)	−0.0027 (7)	0.0064 (7)	0.0130 (7)
O3	0.0240 (7)	0.0338 (8)	0.0318 (7)	0.0074 (6)	−0.0048 (6)	−0.0051 (6)
O4	0.0238 (7)	0.0311 (8)	0.0325 (7)	0.0060 (6)	−0.0054 (6)	−0.0056 (6)
O5	0.0329 (8)	0.0378 (9)	0.0453 (9)	0.0164 (7)	−0.0044 (7)	−0.0066 (7)
O6	0.0456 (9)	0.0340 (8)	0.0293 (7)	0.0036 (7)	0.0066 (6)	0.0025 (6)
O7	0.0269 (7)	0.0305 (8)	0.0350 (8)	0.0057 (6)	−0.0059 (6)	−0.0003 (6)
O8	0.0247 (7)	0.0279 (7)	0.0320 (7)	0.0065 (6)	0.0004 (5)	−0.0014 (6)
N1	0.0208 (8)	0.0267 (8)	0.0255 (8)	0.0011 (6)	−0.0004 (6)	0.0007 (7)
N2	0.0253 (9)	0.0258 (8)	0.0289 (8)	0.0041 (7)	−0.0037 (7)	−0.0022 (7)
N3	0.0201 (8)	0.0247 (8)	0.0312 (8)	0.0039 (6)	−0.0005 (6)	−0.0031 (7)
N4	0.0227 (8)	0.0243 (8)	0.0299 (8)	0.0043 (7)	0.0001 (6)	−0.0042 (7)
C1	0.0342 (11)	0.0307 (11)	0.0338 (11)	0.0055 (9)	0.0019 (8)	−0.0029 (9)
C2	0.0466 (14)	0.0315 (11)	0.0373 (11)	0.0017 (10)	0.0057 (10)	−0.0073 (9)
C3	0.0478 (14)	0.0359 (12)	0.0296 (10)	−0.0062 (10)	0.0019 (9)	−0.0079 (9)
C4	0.0372 (12)	0.0353 (11)	0.0272 (10)	−0.0038 (9)	−0.0034 (8)	0.0015 (9)
C5	0.0280 (10)	0.0247 (9)	0.0234 (9)	−0.0016 (8)	0.0013 (7)	0.0035 (7)
C6	0.0243 (9)	0.0251 (9)	0.0248 (9)	−0.0014 (7)	0.0026 (7)	0.0013 (7)
C7	0.0196 (9)	0.0245 (9)	0.0233 (9)	0.0008 (7)	0.0004 (7)	0.0028 (7)
C8	0.0190 (9)	0.0257 (9)	0.0249 (9)	0.0006 (7)	−0.0008 (7)	0.0014 (7)
C9	0.0225 (9)	0.0230 (9)	0.0244 (9)	0.0000 (7)	0.0015 (7)	0.0015 (7)
C10	0.0273 (10)	0.0226 (9)	0.0271 (9)	−0.0005 (8)	0.0042 (7)	−0.0007 (8)
C11	0.0305 (11)	0.0269 (10)	0.0317 (10)	0.0022 (8)	0.0029 (8)	0.0022 (8)
C12	0.0465 (14)	0.0245 (10)	0.0466 (13)	0.0066 (9)	0.0069 (10)	−0.0024 (9)
C13	0.0544 (15)	0.0286 (11)	0.0394 (12)	−0.0002 (10)	0.0021 (10)	−0.0090 (10)
C14	0.0455 (13)	0.0371 (12)	0.0325 (11)	0.0003 (10)	−0.0038 (9)	−0.0065 (9)
C15	0.0351 (11)	0.0295 (11)	0.0321 (10)	0.0052 (9)	−0.0007 (8)	−0.0028 (9)
C16	0.0458 (13)	0.0359 (12)	0.0283 (10)	−0.0002 (10)	−0.0051 (9)	−0.0002 (9)
C17	0.0616 (16)	0.0451 (14)	0.0274 (11)	−0.0086 (12)	−0.0032 (10)	−0.0099 (10)
C18	0.0577 (16)	0.0365 (12)	0.0356 (12)	−0.0025 (11)	0.0093 (11)	−0.0143 (10)
C19	0.0377 (12)	0.0326 (11)	0.0388 (12)	0.0040 (9)	0.0077 (9)	−0.0066 (9)
C20	0.0283 (10)	0.0269 (10)	0.0247 (9)	0.0014 (8)	0.0040 (7)	−0.0035 (8)
C21	0.0288 (10)	0.0243 (9)	0.0244 (9)	−0.0013 (8)	0.0027 (7)	0.0003 (8)
C22	0.0218 (9)	0.0255 (9)	0.0257 (9)	0.0023 (7)	0.0018 (7)	0.0037 (8)
C23	0.0208 (9)	0.0221 (9)	0.0269 (9)	0.0040 (7)	0.0023 (7)	0.0004 (7)
C24	0.0223 (9)	0.0227 (9)	0.0268 (9)	0.0003 (7)	0.0038 (7)	0.0001 (7)
C25	0.0272 (10)	0.0229 (9)	0.0288 (9)	−0.0032 (8)	0.0075 (7)	−0.0030 (8)
C26	0.0314 (11)	0.0308 (10)	0.0286 (10)	−0.0021 (9)	0.0062 (8)	−0.0015 (8)
C27	0.0475 (14)	0.0412 (13)	0.0306 (11)	−0.0081 (11)	0.0047 (9)	−0.0087 (10)
C28	0.0542 (15)	0.0364 (12)	0.0400 (12)	−0.0059 (11)	0.0127 (11)	−0.0163 (10)
C29	0.0442 (14)	0.0283 (11)	0.0502 (13)	0.0024 (10)	0.0126 (11)	−0.0108 (10)
C30	0.0334 (11)	0.0277 (10)	0.0379 (11)	0.0014 (9)	0.0036 (9)	−0.0035 (9)

### Geometric parameters (Å, °)

**Table:**

Cl1—C11	1.744 (2)	C6—C7	1.473 (3)
Cl2—C26	1.745 (2)	C7—C8	1.362 (3)
S1—O2	1.4284 (15)	C8—C9	1.465 (3)
S1—O1	1.4304 (15)	C10—C15	1.391 (3)
S1—N1	1.6368 (17)	C10—C11	1.401 (3)
S1—C5	1.756 (2)	C11—C12	1.385 (3)
S2—O6	1.4271 (16)	C12—C13	1.384 (3)
S2—O5	1.4302 (15)	C12—H12	0.9500
S2—N3	1.6368 (18)	C13—C14	1.378 (3)
S2—C20	1.761 (2)	C13—H13	0.9500
O3—C7	1.338 (2)	C14—C15	1.389 (3)
O3—H3O	0.83 (3)	C14—H14	0.9500
O4—C9	1.254 (2)	C15—H15	0.9500
O7—C22	1.345 (2)	C16—C17	1.387 (3)
O7—H7O	0.89 (3)	C16—C21	1.392 (3)
O8—C24	1.256 (2)	C16—H16	0.9500
N1—C8	1.435 (2)	C17—C18	1.380 (4)
N1—H1N	0.87 (2)	C17—H17	0.9500
N2—C9	1.346 (2)	C18—C19	1.387 (3)
N2—C10	1.408 (3)	C18—H18	0.9500
N2—H2N	0.82 (2)	C19—C20	1.386 (3)
N3—C23	1.439 (2)	C19—H19	0.9500
N3—H3N	0.89 (2)	C20—C21	1.400 (3)
N4—C24	1.343 (2)	C21—C22	1.474 (3)
N4—C25	1.414 (2)	C22—C23	1.361 (3)
N4—H4N	0.88 (2)	C23—C24	1.467 (3)
C1—C2	1.389 (3)	C25—C26	1.396 (3)
C1—C6	1.394 (3)	C25—C30	1.397 (3)
C1—H1	0.9500	C26—C27	1.384 (3)
C2—C3	1.380 (3)	C27—C28	1.380 (4)
C2—H2	0.9500	C27—H27	0.9500
C3—C4	1.382 (3)	C28—C29	1.385 (4)
C3—H3	0.9500	C28—H28	0.9500
C4—C5	1.387 (3)	C29—C30	1.388 (3)
C4—H4	0.9500	C29—H29	0.9500
C5—C6	1.402 (3)	C30—H30	0.9500
			
O2—S1—O1	119.61 (9)	C10—C11—Cl1	119.75 (16)
O2—S1—N1	107.91 (9)	C13—C12—C11	119.6 (2)
O1—S1—N1	107.08 (9)	C13—C12—H12	120.2
O2—S1—C5	107.76 (10)	C11—C12—H12	120.2
O1—S1—C5	110.81 (9)	C14—C13—C12	119.5 (2)
N1—S1—C5	102.26 (9)	C14—C13—H13	120.3
O6—S2—O5	119.52 (10)	C12—C13—H13	120.3
O6—S2—N3	107.77 (9)	C13—C14—C15	121.4 (2)
O5—S2—N3	107.38 (10)	C13—C14—H14	119.3
O6—S2—C20	108.25 (10)	C15—C14—H14	119.3
O5—S2—C20	110.78 (9)	C14—C15—C10	119.7 (2)
N3—S2—C20	101.59 (9)	C14—C15—H15	120.1
C7—O3—H3O	109.8 (19)	C10—C15—H15	120.1
C22—O7—H7O	107.0 (17)	C17—C16—C21	120.1 (2)
C8—N1—S1	115.68 (13)	C17—C16—H16	119.9
C8—N1—H1N	117.0 (15)	C21—C16—H16	119.9
S1—N1—H1N	110.4 (15)	C18—C17—C16	120.9 (2)
C9—N2—C10	130.09 (17)	C18—C17—H17	119.6
C9—N2—H2N	113.3 (17)	C16—C17—H17	119.6
C10—N2—H2N	116.5 (17)	C17—C18—C19	120.3 (2)
C23—N3—S2	115.79 (13)	C17—C18—H18	119.9
C23—N3—H3N	115.3 (15)	C19—C18—H18	119.9
S2—N3—H3N	111.6 (15)	C20—C19—C18	118.6 (2)
C24—N4—C25	130.44 (17)	C20—C19—H19	120.7
C24—N4—H4N	116.0 (15)	C18—C19—H19	120.7
C25—N4—H4N	113.4 (15)	C19—C20—C21	122.03 (19)
C2—C1—C6	120.2 (2)	C19—C20—S2	120.40 (16)
C2—C1—H1	119.9	C21—C20—S2	117.38 (15)
C6—C1—H1	119.9	C16—C21—C20	118.09 (19)
C3—C2—C1	120.6 (2)	C16—C21—C22	120.74 (19)
C3—C2—H2	119.7	C20—C21—C22	121.17 (17)
C1—C2—H2	119.7	O7—C22—C23	123.13 (18)
C2—C3—C4	120.3 (2)	O7—C22—C21	114.48 (17)
C2—C3—H3	119.8	C23—C22—C21	122.39 (17)
C4—C3—H3	119.8	C22—C23—N3	120.84 (17)
C3—C4—C5	119.1 (2)	C22—C23—C24	121.70 (17)
C3—C4—H4	120.5	N3—C23—C24	117.32 (16)
C5—C4—H4	120.5	O8—C24—N4	124.42 (18)
C4—C5—C6	121.64 (19)	O8—C24—C23	120.82 (17)
C4—C5—S1	120.83 (16)	N4—C24—C23	114.76 (16)
C6—C5—S1	117.38 (15)	C26—C25—C30	118.66 (19)
C1—C6—C5	118.06 (18)	C26—C25—N4	117.81 (18)
C1—C6—C7	120.92 (18)	C30—C25—N4	123.53 (19)
C5—C6—C7	121.01 (17)	C27—C26—C25	121.4 (2)
O3—C7—C8	123.19 (17)	C27—C26—Cl2	118.85 (17)
O3—C7—C6	114.07 (16)	C25—C26—Cl2	119.75 (16)
C8—C7—C6	122.73 (17)	C28—C27—C26	119.5 (2)
C7—C8—N1	120.91 (17)	C28—C27—H27	120.3
C7—C8—C9	121.83 (17)	C26—C27—H27	120.3
N1—C8—C9	117.07 (16)	C27—C28—C29	119.9 (2)
O4—C9—N2	123.81 (18)	C27—C28—H28	120.0
O4—C9—C8	120.71 (17)	C29—C28—H28	120.0
N2—C9—C8	115.45 (17)	C28—C29—C30	121.0 (2)
C15—C10—C11	118.37 (18)	C28—C29—H29	119.5
C15—C10—N2	124.07 (18)	C30—C29—H29	119.5
C11—C10—N2	117.55 (18)	C29—C30—C25	119.6 (2)
C12—C11—C10	121.4 (2)	C29—C30—H30	120.2
C12—C11—Cl1	118.89 (17)	C25—C30—H30	120.2
			
O2—S1—N1—C8	−63.66 (16)	C13—C14—C15—C10	0.8 (4)
O1—S1—N1—C8	166.37 (14)	C11—C10—C15—C14	0.1 (3)
C5—S1—N1—C8	49.81 (16)	N2—C10—C15—C14	−178.5 (2)
O6—S2—N3—C23	−63.06 (16)	C21—C16—C17—C18	0.3 (4)
O5—S2—N3—C23	166.96 (14)	C16—C17—C18—C19	0.9 (4)
C20—S2—N3—C23	50.61 (16)	C17—C18—C19—C20	−1.0 (4)
C6—C1—C2—C3	0.0 (3)	C18—C19—C20—C21	0.1 (3)
C1—C2—C3—C4	1.4 (4)	C18—C19—C20—S2	175.04 (18)
C2—C3—C4—C5	−0.5 (3)	O6—S2—C20—C19	−97.28 (19)
C3—C4—C5—C6	−1.7 (3)	O5—S2—C20—C19	35.6 (2)
C3—C4—C5—S1	173.86 (16)	N3—S2—C20—C19	149.40 (18)
O2—S1—C5—C4	−96.73 (18)	O6—S2—C20—C21	77.92 (18)
O1—S1—C5—C4	35.9 (2)	O5—S2—C20—C21	−149.22 (16)
N1—S1—C5—C4	149.69 (17)	N3—S2—C20—C21	−35.39 (18)
O2—S1—C5—C6	78.99 (17)	C17—C16—C21—C20	−1.2 (3)
O1—S1—C5—C6	−148.42 (15)	C17—C16—C21—C22	178.5 (2)
N1—S1—C5—C6	−34.59 (17)	C19—C20—C21—C16	1.1 (3)
C2—C1—C6—C5	−2.2 (3)	S2—C20—C21—C16	−174.06 (16)
C2—C1—C6—C7	179.0 (2)	C19—C20—C21—C22	−178.64 (19)
C4—C5—C6—C1	3.0 (3)	S2—C20—C21—C22	6.2 (3)
S1—C5—C6—C1	−172.66 (15)	C16—C21—C22—O7	16.3 (3)
C4—C5—C6—C7	−178.18 (18)	C20—C21—C22—O7	−163.96 (18)
S1—C5—C6—C7	6.1 (2)	C16—C21—C22—C23	−164.7 (2)
C1—C6—C7—O3	13.8 (3)	C20—C21—C22—C23	15.0 (3)
C5—C6—C7—O3	−164.97 (17)	O7—C22—C23—N3	−179.88 (17)
C1—C6—C7—C8	−167.18 (19)	C21—C22—C23—N3	1.2 (3)
C5—C6—C7—C8	14.1 (3)	O7—C22—C23—C24	4.5 (3)
O3—C7—C8—N1	−178.75 (17)	C21—C22—C23—C24	−174.39 (17)
C6—C7—C8—N1	2.3 (3)	S2—N3—C23—C22	−38.4 (2)
O3—C7—C8—C9	6.4 (3)	S2—N3—C23—C24	137.43 (15)
C6—C7—C8—C9	−172.57 (17)	C25—N4—C24—O8	1.1 (3)
S1—N1—C8—C7	−38.3 (2)	C25—N4—C24—C23	−177.98 (18)
S1—N1—C8—C9	136.81 (15)	C22—C23—C24—O8	−8.3 (3)
C10—N2—C9—O4	−2.1 (3)	N3—C23—C24—O8	175.95 (17)
C10—N2—C9—C8	179.71 (19)	C22—C23—C24—N4	170.83 (18)
C7—C8—C9—O4	−9.2 (3)	N3—C23—C24—N4	−4.9 (3)
N1—C8—C9—O4	175.77 (17)	C24—N4—C25—C26	169.9 (2)
C7—C8—C9—N2	169.08 (18)	C24—N4—C25—C30	−10.4 (3)
N1—C8—C9—N2	−6.0 (3)	C30—C25—C26—C27	0.6 (3)
C9—N2—C10—C15	−10.1 (3)	N4—C25—C26—C27	−179.67 (19)
C9—N2—C10—C11	171.3 (2)	C30—C25—C26—Cl2	−179.24 (16)
C15—C10—C11—C12	−0.9 (3)	N4—C25—C26—Cl2	0.5 (3)
N2—C10—C11—C12	177.8 (2)	C25—C26—C27—C28	0.1 (3)
C15—C10—C11—Cl1	179.31 (16)	Cl2—C26—C27—C28	−179.99 (18)
N2—C10—C11—Cl1	−2.0 (3)	C26—C27—C28—C29	−0.7 (4)
C10—C11—C12—C13	0.8 (3)	C27—C28—C29—C30	0.5 (4)
Cl1—C11—C12—C13	−179.42 (19)	C28—C29—C30—C25	0.2 (3)
C11—C12—C13—C14	0.1 (4)	C26—C25—C30—C29	−0.8 (3)
C12—C13—C14—C15	−0.9 (4)	N4—C25—C30—C29	179.5 (2)

### Hydrogen-bond geometry (Å, °)

**Table:**

*D*—H···*A*	*D*—H	H···*A*	*D*···*A*	*D*—H···*A*
O3—H3O···O4	0.83 (3)	1.89 (3)	2.612 (2)	144 (3)
O3—H3O···O4^i^	0.83 (3)	2.33 (3)	2.854 (2)	122 (2)
O7—H7O···O8	0.89 (3)	1.81 (3)	2.607 (2)	147 (2)
O7—H7O···O8^ii^	0.89 (3)	2.46 (3)	2.964 (2)	116 (2)
N1—H1N···O8^ii^	0.87 (2)	2.06 (2)	2.911 (2)	164 (2)
N2—H2N···N1	0.82 (2)	2.24 (2)	2.700 (2)	116 (2)
N2—H2N···Cl1	0.82 (2)	2.47 (2)	2.930 (2)	116 (2)
N3—H3N···O4^i^	0.89 (2)	2.07 (2)	2.912 (2)	157 (2)
N4—H4N···N3	0.88 (2)	2.23 (2)	2.692 (2)	113 (2)
N4—H4N···Cl2	0.88 (2)	2.41 (2)	2.934 (2)	119 (2)

Symmetry codes: (i) −*x*+2, −*y*+1, −*z*+1; (ii) −*x*+1, −*y*+1, −*z*+1.
